# Myasthenia gravis complicated with autoimmune encephalitis: a review

**DOI:** 10.3389/fneur.2026.1812305

**Published:** 2026-04-24

**Authors:** Nils Erik Gilhus

**Affiliations:** 1 Department of Clinical Medicine, University of Bergen, Bergen, Norway; 2 Department of Neurology, Haukeland University Hospital, Bergen, Norway

**Keywords:** autoantibodies, autoimmune disease, autoimmune encephalitis, comorbidity, FcRn-blockers, intravenous immunoglobulin, myasthenia gravis

## Abstract

**Objective:**

This review aims to provide updated information about myasthenia gravis (MG) complicated with autoimmune encephalitis.

**Background:**

MG and autoimmune encephalitis are both antibody-mediated disorders with a need for active and individually adapted immunosuppressive treatment. The two disorders can co-exist. This represents therapeutic and diagnostic challenges but may help in elucidating disease mechanisms and risk factors.

**Methods:**

A literature search combined the words “myasthenia gravis,” “autoimmune encephalitis,” and further combined each specific antibody associated with autoimmune encephalitis and “myasthenia gravis.”

**Results:**

Epidemiological data and multiple single case reports illustrate the increased risk for autoimmune encephalitis in MG. The target antigens for the antibodies in both MG and autoimmune encephalitis vary among patients. Clinical manifestations, choice of therapy, and prognosis depend on antibody pattern. MG should be treated actively with immunosuppression according to updated and generally accepted guidelines. For treatment of autoimmune encephalitis, high corticosteroid doses, intravenous immunoglobulin (IVIg), plasma exchange, and rituximab should be considered. FcRn-blockers represent an additional option.

**Conclusion:**

MG and autoimmune encephalitis have overlapping pathogenesis and similar preferred drug therapies. This comorbidity represents a therapeutic challenge, and often with the need of high-dose immunosuppressive combination therapy. New and targeted therapies are applied in some MG patients, and such therapies have relevance also for autoimmune encephalitis.

## 1 Introduction

Myasthenia gravis (MG) is an autoantibody-mediated disease with an immune attack against the postsynaptic membrane at the neuromuscular junction and muscle weakness as the dominating manifestation. The disease is subgrouped based on clinical and pathogenetic biomarkers including symptom-inducing antibody, thymus pathology, age at symptom debut, and disease generalization (1). Acetylcholine receptor (AChR) antibodies can be detected in 85% of MG patients, whereas antibodies against muscle-specific kinase (MuSK) are found in 1–10% depending on population and geography (2). Lipoprotein-related protein 4 (LRP4) is the target for antibodies in 1–2%, whereas 10% of MG patients remain seronegative with no detectable antibodies against antigens at the neuromuscular junction. MG prevalence increases worldwide and for all subgroups, most for the very old patients with AChR antibodies (3).

Comorbidities are common in MG, especially in the older age group and those with a thymoma (4). They are important for choice of therapy, therapeutic response, and prognosis. Comorbidities may help in revealing disease mechanisms and etiology, including autoimmune overlaps. Comorbidities may be difficult to diagnose, as the typical MG symptoms of muscle weakness, fatigue, and impaired activities of daily life are non-specific and can be attributed to several disorders.

Autoimmune encephalitis is associated with a range of autoantibodies, some being disease-inducing (5). The symptoms vary, but characteristic features are mental change, reduced consciousness, and focal epilepsy. The disorders can be classified from clinical manifestations and immunological target (6). They can be paraneoplastic, post-infectious, and idiopathic. Whereas therapy in MG is based on controlled studies, well-characterized real-life cohorts, and unified guidelines (7–10), this is not so for autoimmune encephalitis. The condition is rare and with great variation in antibody-pattern. Treatment tends to be based on single case reports, small patient series, and consensus documents.

The co-occurrence of MG and autoimmune encephalitis can shed light on autoimmune disease pathogenesis. New and emerging MG therapies may turn out to be effective also for autoimmune encephalitis. Awareness of the increased co-occurrence should help in an early diagnosis of autoimmune encephalitis in MG patients. Paraneoplasia and risks associated with immune checkpoint inhibitors are relevant for both disorders. This review gives an update on epidemiology, pathogenesis, and diagnostic challenges and recommends active combination therapy for both disorders.

## 2 Methods

The evidence was collected by the author searching the Web of Science database (webofscience.com) on October 27th, 2025, with the combined key words “myasthenia gravis” and “autoimmune encephalitis.” The search was limited to publications in English. No limitations regarding document type or year of publication were introduced. The search resulted in 211 hits, 95 being listed as clinical neurology, 65 neuroscience, 51 immunology, 22 pharmacology, 15 oncology, and 8 psychology. From the title and abstract, 30 articles regarded by the author to be of special interest were selected and retrieved in full text. Additional articles were included based on the reference list of the selected publications as well as based on the author’s general knowledge of the field. Specific searches were made for relevant topics not fully answered by articles identified by the systematic review. Further searches were undertaken on November 25^th^, 2025, combining the search word “myasthenia gravis” with each of the specific antibody names as listed in Table 1. Another search combining “myasthenia gravis” and “autoimmune encephalitis” was performed on January 27^th^, 2026. Among the 10 new articles listed, five were retrieved in full text. The Cochrane database was searched for “myasthenia gravis.” None of the 12 hits were relevant for this review article. The Pubmed database was searched for the combination “myasthenia gravis” and “autoimmune encephalitis.” There were 7 hits for both search words in the title, 17 hits for “myasthenia gravis” in the title and “autoimmune encephalitis” in the abstract, and 91 hits with both search words in the abstract. All these abstracts were checked for relevance, nearly all overlapping with the primary Web of Science search.

**Table 1:** Brain antigens for the antibodies in autoimmune encephalitis and their matching phenotype, as reviewed by de Bruijn et al. (5).

Antigen	Phenotype	MG association
Hu	Limbic and brainstem encephalitis	−
Yo	Cerebellar degeneration	−
Ma1	Limbic and brainstem encephalitis	−
Ma2	Brainstem and limbic encephalitis	(+)
CRMP5	Encephalomyelitis	(+)
Amphiphysin	Limbic encephalitis. Stiffperson syndrome.	−
Ri	Brainstem encephalitis	−
Tr	Cerebellar degeneration	−
KLHL11	Brainstem and limbic encephalitis. Cerebellar degeneration	−
NMDAR	Encephalitis with psychosis, memory loss, dyskinesia, sleep disturbance	+
LGI1	Limbic encephalitis	++
CASPR2	Encephalitis. Morwan’s syndrome	+++
GABA_B_	Limbic encephalitis, prominent seizures	+
GABA_A_	Encephalitis, prominent seizures	+
AMPAR	Limbic encephalitis	++(+)
GlyR	Encephalomyelitis with rigidity and myoclonus	+
mGluR5	Encephalitis	−
DPPX	Encephalitis	−
AK5	Limbic encephalitis	−
IgLON5	Encephalitis with parasomnia	−
GFAP	Meningoencephalitis	−
GAD65	Limbic encephalitis, refractory seizures	+
MOG	Cortical encephalitis	+

Risk of association with myasthenia gravis (MG) was graded subjectively by the author from – to +++ based on reported cases and reviews. +++ indicates a much higher association than expected by chance, + and ++ both indicate a more common co-occurrence than just by chance, whereas (+) and − indicate no proven association, could be by chance. Precise incidence estimates were not possible to generate.

## 3 Results

### 3.1 Epidemiology

The combination of MG and autoimmune encephalitis is rare. The largest patient series included 18 cases, all with a thymoma (11). More than 15 reports of a single patient with the combination have been published. Patients with this combination also appear in small case series, in reports of various autoimmune comorbidities, and in reviews. Some of these do not represent unique cases. Thymoma-associated MG, autoimmune encephalitis, and CASPR2- antibodies repressnt the most common combination.

MG has an overall prevalence of around 25 per 100,000, and an annual incidence of around 20 per one million (3). Both prevalence and incidence figures are highest in the elderly group, an annual incidence of 63 per million being reported above age 65 years (12). The prevalence has increased markedly during the last decades. Also, incidence has increased, in part because of increased case-finding (13). Recent Danish registry data strongly indicate a true increase in MG incidence among the very old (14).

Precise epidemiological data for autoimmune encephalitis is lacking. The disorder is rare in all age groups. An annual incidence of 8 per million was reported from Olmsted County 1995–2015, with a prevalence of 14 per million (15). Autoimmune anti-N-methyl-D-aspartate (NMDA) receptor encephalitis is responsible for up to 40% of all seropositive cases, with female and young age preponderance (16). Overall annual incidence of autoimmune encephalitis in children ≤ 18 years was 1.5 per million (17). A Dutch nationwide study reported an annual incidence of autoimmune paraneoplastic encephalitis of 5.6 per million, and with a marked increase during the recording period 2016–2021 (18).

If MG and autoimmune encephalitis were unrelated disorders, one would expect the co-occurrence in 1 in 3.5 × 10^9^ individuals based on prevalences of 250 and 8 per million, respectively. The number of reported cases clearly illustrates that there are common risk factors. Thymoma is the strongest risk factor for the combination (19). Whereas immunological senescence explains an increased MG risk in the very old (20), epidemiological data does not support this as a risk factor for the combination of MG and autoimmune encephalitis.

### 3.2 Disease manifestations

MG is a fluctuating disease. The muscle weakness in most patients is mild to moderate, but with a risk of exacerbations. Even with novel treatments around 10% of MG patients are regarded as difficult to treat with highly insufficient treatment response (21).10–20% of MG patients experience during their life-time a myasthenic crisis with the need of respiratory support (22, 23). Complete MG remission is rare and occurs mainly when MG onset in childhood (24). Full remission occurred even more rarely with thyroid antibodies. That might be true for autoimmune comorbidities in general. Pharmacological remission or minimal symptom expression should be the treatment aim for all MG patients (8, 25, 26).

The risk for autoimmune encephalitis in an MG patient does not depend on MG symptom severity. There is no increased risk for autoimmune encephalitis during MG exacerbation or crisis. Autoimmune encephalitis can appear in MG patients who have been in a stable remission for many years (27), and it can appear in MG with purely ocular weakness (28). The severity of the encephalitis is not associated with the severity of the MG, and the presence of another autoimmune disease does not affect the progression or clinical outcome of the autoimmune encephalitis (29). During autoimmune encephalitis the MG usually remains unchanged and stable.

### 3.3 Pathogenesis

In 85% of MG patients, AChR antibodies, mainly of the IgG1 and IgG3 subclass, bind to AChR in the postsynaptic membrane (2). This leads to complement activation with destruction of the membrane, cross-binding of AChR with increased receptor degradation, and blocking of acetylcholine binding sites. Compensatory mechanisms involve increased synthesis of new AChR and active repair of the postsynaptic membrane at the neuromuscular junction (30). MG should be subgrouped to support therapeutic decisions (Table 2), and juvenile and very late onset have been added as new and distinct subgroups.

**Table 2:** Myasthenia gravis subgroups relevant for optimizing therapy and estimating risk for autoimmune encephalitis (AE).

Subgroup	Antibody	Age at onset	Thymus	AE association
Juvenile	AChR	<18 years	Hyperplasia	+(+)
Early onset	AChR	18–50 years	Hyperplasia	++
Late onset	AChR	50–65 years	Variable	+
Very late onset	AChR	> 65 years	Normal	+
Thymoma	AChR	Any	Tumour	+++
Muscle-specific kinase	MuSK	Any	Normal	(+)
LRP4	LRP4	Any	Normal	(+)
Ocular	Variable	Any	Variable	+
Seronegative	None	Any	Normal	(+)

AE association was graded by the author from (+) to +++ based on reported cases and reviews. +++ indicates a much higher association than expected by chance, + and ++ both indicate a more common co-occurrence than just by chance, whereas (+) indicates no or unknown association. Precise incidence estimates were not possible to generate.

In 10–15% of MG patients, a thymoma initiates the production of AChR antibodies. Muscle-like epitopes on the thymoma cells are presented for developing T-lymphocytes and the intrathymic negative selection process fails (31). In these patients. MG represents a paraneoplastic disorder.

Most juvenile and early onset MG patients (debut age < 50 years) have thymic hyperplasia with germinal follicles and activated B- and T-lymphocytes. The sensitization against AChR is caused by aberrant presentation of muscle-like epitopes within thymus, leading to failure of negative selection of specific T-lymphocytes (32). These T cells are exported to the peripheral lymphoid tissue where they stimulate B-lymphocytes, plasma cells, and plasmablasts to AChR antibody production. A minority of late onset MG patients (debut age 50–65 years) have thymic hyperplasia (33), whereas no pathogenic involvement of thymus has been shown in very late onset MG.

In 1–10% of MG patients, circulating antibodies against the postsynaptic membrane protein MuSK is found instead of AChR antibodies (2). MuSK antibodies are mainly of the IgG4 subclass (34). They do not activate complement and they do not promote inflammation. Their *in vivo* binding to MuSK impairs the physiological clustering of AChR in the postsynaptic membrane, reduces AChR function, and impairs neuromuscular signal transmission (35). MG patients with MuSK antibodies have no thymus abnormalities. A small MG minority have antibodies against the muscle membrane protein LRP4 (36).

In the remaining MG patients, no pathogenic muscle antibodies can be detected. The pathogenesis in this group is probably heterogeneous, with some patients having low-concentration and low-affinity antibodies against AChR, MuSK, or LRP4, some having antibodies against other postsynaptic muscle membrane molecules, and some having a non-muscle-antibody mediated disease.

MG is caused by the combination of predisposing genes and unknown environmental factors. In paraneoplasia, a thymoma represents this external factor. In early and late onset MG with AChR antibodies, an infection has been speculated to trigger the autoimmune response. The hyperplastic thymus has been extensively examined for signs of ongoing or previous infection, but no infection has been convincingly confirmed. The individual immunological response to an infection is most probably more important than the infectious particle itself for any development of MG years later. In very old onset MG, immunosenescence is probably a causative factor (20).

Autoimmune encephalitis represents a heterogeneous disorder where detailed knowledge about pathogenesis helps in subgrouping of patients and in finding optimal therapy (Table 1). Similarities to MG pathogenesis explain why autoimmune encephalitis appears more often in MG than just by chance and illustrate the possibility of joint therapeutic interventions. Autoantibodies are markers for autoimmune encephalitis. They can be directed against cell membranes or intracellular brain antigens (5). Encephalitis with antibodies against intracellular antigens is usually paraneoplastic. Such antibodies are not causing the symptoms as intact cell membranes protect against antibody penetration. Still, there is evidence of novel mechanisms through which antibodies against intracellular antigens may exert pathogenic effects. Autoimmune encephalitis with antibodies to intracellular antigens is only very rarely associated with MG (37, 38).

Autoimmune encephalitis with antibodies against neuronal surface antigens in the brain is associated with MG (Table 1). Among 507 cases with thymoma and a paraneoplastic disease, 63% had MG and 6% had autoimmune encephalitis (19). All thymoma MG patients have AChR antibodies. Those with autoimmune encephalitis have in addition antibodies with various specificities against neuronal cell membrane antigens (11). One third of patients with thymoma-associated autoimmune encephalitis but no MG had antibodies against intracellular antigens (39). Most of them had in addition antibodies against cell membrane antigens. The onset of MG is usually several months or years before the autoimmune encephalitis, but encephalitis preceding the MG in thymoma patients has been reported (11). The MG characteristics in those with autoimmune encephalitis do not differ from other thymoma-MG cases.

Among non-thymoma MG, autoimmune encephalitis is more common with the early onset than the late and very late onset groups (38) (Table 2). All autoimmune comorbidity is more common in the young MG subgroup (40). This may be explained by joint genetic predisposition and perhaps also the thymus pathology in early onset MG.

Non-motor MG symptoms can include autonomic disorders, cognitive impairment, sleep disturbance, and psychological problems (41, 42). Comorbid autoimmune encephalitis with central nervous system antibodies is one among several potential mechanisms for such symptoms (41).

Several MG-susceptibility gene loci have been identified, not least by GWAS studies (43, 44). Epigenetic mechanisms including DNA methylation, histone modification, and non-coding RNAs all influence MG risk. The specific genetic risk factors vary between MG subgroups. HLA-B*08.01 represents a primary risk factor for early onset MG with AChR antibodies, whereas HLA-DRB1*15.01 is associated with late onset MG (45). MuSK-MG is associated with the HLA-DR14-DQ5 haplotype, being relevant for the IgG4 immune response (43, 46). Thymoma MG patients have a separate HLA profile (43). Several non-HLA genes being relevant for immune reactivity are associated with MG and MG subgroups (44). Family studies confirm that up to half of the MG risk can be explained by genetic predisposition (3). Transcriptome-wide association studies examine gene expression and such studies should further elucidate MG pathogenesis (47).

Gene associations can be linked to autoimmune diseases in general and to autoimmune diseases mediated by autoantibodies. The HLA-DRB1*16.02 allele is associated with many autoantibody-mediated disorders including both MG and subtypes of autoimmune encephalitis, but not with T-cell mediated autoimmune disorders (48). MG is genetically correlated to most other autoimmune disorders (44). Risk loci have been identified for autoimmune encephalitis subgroups. Both HLA alleles and genes outside HLA are associated with autoimmune encephalitis and antibodies to leucine-rich glioma-inactivated 1 (LGI1) (49). The genetic profile influences key clinical aspects such as debut age, severity, and response to therapy (50). Autoimmune encephalitis with antibodies to the N-methyl-D-aspartate receptor (NMDAR) has a genetic susceptibility linked to both HLA and non-HLA genes (51). The overlap with MG-associated genes for NMDAR encephalitis is very limited, in line with only minimal clinical association (Table 1).

MG patients can develop autoimmune encephalitis when on immunosuppressive treatment, and even in high doses. This may be seen as a paradox as such treatment might have a protective effect. However, the causative and contributory factors common for MG and autoimmune encephalitis in some individuals override such protection. Without immunosuppressive treatment the risk for autoimmune comorbidity would probably have been higher.

### 3.4 Autoantibodies

AChR-, MuSK-, and LRP-antibodies induce muscle weakness in MG. Antibody concentration does not reflect MG severity, but changes over time has some value in predicting disease development and prognosis (52, 53). Many MG patients have additional antibodies against other muscle antigens, including titin, ryanodine receptor, agrin, cortactin, and collagen Q (54, 55). Antibodies to such intracellular targets are probably not pathogenic. However, some of them have diagnostic and prognostic value (56, 57). AChR antibodies directed against the fetal subunit (*γ*-unit) can influence motor development in children of MG mothers during pregnancy and lead to fetal acetylcholine receptor inactivation syndrome (58, 59). Muscle antibodies do not appear regularly with autoimmune encephalitis. There is no particular muscle antibody pattern that is associated with autoimmune encephalitis, but this comorbidity is rarely reported in MuSK MG, LRP4 MG, and seronegative MG (Table 2).

Autoantibody testing plays a crucial role in diagnosing autoimmune encephalitis, and autoimmune encephalitis is grouped according to disease-inducing antibody (5). Most antibody tests are performed with commercially available kits. Diagnostic accuracy is improved by testing both serum and cerebrospinal fluid. The key antibodies in autoimmune encephalitis have recently been summarized by de Bruijn et al. (5) (Table 1). In a large patient series, autoimmune comorbidities were more common with anti-LGI1 than with anti-NMDAR encephalitis (29). In a systematic review, 38 patients with CASPR2 antibody syndrome had comorbid MG, and MG was the most common comorbid neurological association for autoimmune encephalitis patients with this antibody (60).

AChR antibodies are mostly of the IgG1 and IgG3 subclass, as are most antibodies in autoimmune disorders (2). In contrast, MuSK MG patients have antibodies of the IgG4 subclass. In several patients with LGI1, CASPR2, IgLON5, and DPXX antibodies, the antibodies are of the IgG4 subclass (61). Such antibodies modulate function but do not induce inflammation (62, 63).

### 3.5 Immune checkpoint inhibitors

Immune checkpoint inhibitors have improved the survival for patients with many solid organ cancers. Due to their mechanism of action they can induce autoimmune disorders (64). Neurological adverse effects are reported in 1–5% of cancer patients treated with immune checkpoint inhibitors. MG and encephalitis are among the most common autoimmune manifestations (65). Complications from the muscle and brain represent a major cause of intensive care unit admissions in patients treated with checkpoint inhibitors (66). The MG is often severe and difficult to treat, with progressive and generalized muscle weakness. Myositis is a common complicating feature. Most, but not all, have AChR antibodies (67). Some patients have antibodies against other muscle antigens such as MuSK, titin, and ryanodine receptor. Mean onset time for the neurological, immune-mediated side-effects after start of checkpoint inhibitor treatment is 30 days (64). In a recent study, 12/18 MG patients had a deterioration when treated with immune checkpoint inhibitors, six needed hospitalization, and two of them died from their MG (68).

Immune checkpoint inhibitors can induce powerful anti-tumor responses, but via the same mechanisms induce immune-related side-effects (67). T cells normally protect against neoplasms. This protection can be inhibited by regulatory mechanisms induced by the tumor cells. The checkpoint inhibitors can block this inhibition of immune protection, leading to activation of T cells, and in a small minority of patients to new autoimmune disease. The function of B cells is also influenced by checkpoint inhibitors, contributing to the risk for both MG and autoimmune encephalitis (67). Limbic encephalitis, meningoencephalitis, and cerebellitis are phenotypes associated with checkpoint inhibitors (69), and with the same autoantibodies that are found in paraneoplastic autoimmune encephalitis (67). This indicates a common immune pathogenesis (70).

Routine screening for muscle and neuronal antibodies should be considered before starting treatment with checkpoint inhibitors. The presence of such antibodies increases the risk for developing autoimmune disease including MG and autoimmune encephalitis (67). The fact that immune checkpoint inhibitors increase the risk for MG and autoimmune encephalitis more than for most other autoimmune disorders illustrates that these two disorders have pathogenic overlaps.

MG and autoimmune encephalitis induced by immune checkpoint inhibitors are difficult to treat. Early and active immunosuppression is most important (67, 71). This means fast-acting drugs in high concentrations. The same drugs as for severe MG not induced by checkpoint inhibitors are generally recommended. In addition to corticosteroids, IVIg, and plasma exchange, successful treatment with intravenous methotrexate, rituximab, and efgartigmod have been reported (72, 73). Treatment of the underlying cancer is crucial for patient survival and total outcome.

### 3.6 Therapy

Most MG patients need active immunosuppressive treatment. The treatment aims should be ambitious. Minimal symptom expression can be obtained in many patients, and even pharmacological remission. The treatment should consider that MG is a chronic but fluctuating disease. The immunosuppressive treatment is not MG-specific, i.e., not specifically targeted toward the AChR or MuSK autoimmune response. Thus, the immunosuppressive treatment in MG is highly relevant also for autoimmune encephalitis.

In patients with autoimmune encephalitis, the response to immunosuppression is variable. The great distinction is between patients with antibodies against intracellular and cell membrane antigens (6). Furthermore, autoimmune encephalitis is usually a unipolar disease, and the treatment duration is limited. Due to the rarity and heterogeneity of autoimmune encephalitis the type and length of immunotherapy is empiric and not based on controlled studies. For the combination with MG, only a limited number of case reports exist.

MG treatment is based on international and national guidelines, some controlled studies, but hardly well-controlled studies that compare different treatment regimens (7–10). Corticosteroids in the lowest possible dose combined with azathioprine, mycophenolate, or tacrolimus represent first-line treatments. Rituximab, an anti CD20 B-cell monoclonal antibody, is an alternative initial treatment, especially with MuSK-antibodies (25), but also with AChR antibodies (7, 9). For acute and subacute exacerbations and myasthenic crisis, intravenous immunoglobulin (IVIg) and plasma exchange are the two preferred treatments. Complement inhibitors and FcRn blockers have in controlled studies shown a rapid and marked effect in patients with AChR antibodies who have been difficult to treat (8). FcRn blockers have a good effect in MuSK-MG. An anti-CD19 B-cell antibody had a clear positive effect in MG patients in a recent controlled study (74). Chimeric antigen receptor cell (CAR-T) therapy targets pathogenic immune cell populations more specifically (75). Such therapy is promising for autoimmune disorders and has proven to be effective in MG (76, 77). Experimental studies for autoimmune encephalitis indicate positive results but clinical trials are needed.

For autoimmune encephalitis with antibodies against the neuronal cell membrane or synaptic proteins, corticosteroids, IVIg, and plasma exchange represent first-line therapies (5, 78). The second-line therapies similarly consist of drugs recommended for MG and include rituximab, azathioprine, mycophenolate mofetil, and cyclophosphamide. Removal of the tumour is crucial for neoplastic disorders, and this includes a thymoma in MG. Supportive therapy is important both for autoimmune encephalitis and MG.

Monoclonal antibodies targeting B cells, FcRn, and the complement cascade have been suggested as potential therapies for autoimmune encephalitis (79). Rituximab represents an established therapy (80). The evidence for effect is best for NMDAR-encephalitis, but also patients with other neuronal autoantibodies respond. Ocrelizumab, inebilizumab, and daratumumab are monoclonal antibodies directed against CD20, CD19, and CD38 on B cells, respectively. There are single case reports of a positive effect in otherwise therapy-resistant autoimmune encephalitis (78), but no reported formal trial results. Bortezomid, a proteasome inhibitor depleting plasma cells, has been reported to have a positive effect in single patients with aggressive autoimmune encephalitis and various types of antibodies, and also in some MG patients (81). It might be best suited for NMDAR encephalitis. FcRn blockers inhibit the recycling of IgG but not of any other immunoglobulin classes. They are expected to have a therapeutic effect in IgG-autoantibody-mediated disorders, thus including some patients with autoimmune encephalitis. In a series of 17 patients, efgartigimod gave significant clinical improvement (82). In a case–control study of NMDAR encephalitis, treatment with efgartigimod and IVIg gave similar symptom improvement (83). Efgartigimod In combination with intravenous methylprednisolone gave rapid and marked improvement in a retrospective study including 122 patients with antibody-mediated autoimmune encephalitis (84). Successful treatment with efgartigimod has been reported in a MG patient with anti-GQ1b autoimmune encephalitis (85). There are several single cases of autoimmune encephalitis with various autoantibodies and a positive effect of an FcRn blocker (86).

Evidence for complement-mediated neuronal toxicity in autoimmune encephalitis is lacking. However, IgG antibodies apart from IgG4 bind complement, and complement inhibitors may have a therapeutic effect in some patients with autoimmune encephalitis, especially with CASPR2 and LGI1 antibodies (79, 87).

Patients with the combination of MG and autoimmune encephalitis should continue or intensify their standard MG immunosuppressive treatment. The corticosteroid dose should usually be increased to treat the encephalitis more effectively. IVIg or plasma exchange is recommended as additional therapy for most patients with autoimmune encephalitis. If insufficient or no response, a FcRn blocker may be tried when the antibodies are directed against cell membrane antigens as FcRn blockers are reported to have an additional effect in uncontrolled studies of a limited number of patients.

## 4 Conclusion

MG and autoimmune encephalitis are rare, antibody-mediated disorders. Epidemiological data show that the risk of autoimmune encephalitis is increased in MG. The co-occurrence of these two disorders illustrates pathogenic similarities (88, 89). Autoimmune overlap is in part due to predisposing genes, especially in the HLA-system. MG and autoimmune encephalitis might also have shared muscular and neuronal antigen epitopes, although evidence for this is lacking. Dysregulation within a thymoma leading to failure to suppress or inhibit export of self-reactive T lymphocytes may be a joint mechanism, involving some but not all T cells with self-antigen specificities (32). Both MG and autoimmune encephalitis should be subgrouped according to autoantibody pattern as clinical manifestations, prognosis, and optimal therapy depend on this pattern. Autoimmune encephalitis should be suspected in MG patients who develop memory deficits, altered mental status, psychiatric symptoms, or epileptic seizures. Autoimmune encephalitis should be treated early and active with high corticosteroid doses, IVIg or plasma exchange, rituximab, and FcRn blocker in treatment-resistant patients. Most MG-associated encephalitis patients have antibodies against cell-membrane and synaptic proteins, and therefore a therapeutic response is expected. Active immunosuppressive treatment should benefit the MG as well.
